# Phosphoproteomics reveals that cinobufotalin promotes intrahepatic cholangiocarcinoma cell apoptosis by activating the ATM/CHK2/p53 signaling pathway

**DOI:** 10.3389/fonc.2022.982961

**Published:** 2022-09-16

**Authors:** Zhili Xia, Minzhen Li, Meng Hu, Yanyan Lin, Lawrence Lawer Atteh, Wenkang Fu, Long Gao, Mingzhen Bai, Chongfei Huang, Ping Yue, Yu Liu, Wenbo Meng

**Affiliations:** ^1^The First Clinical Medical College, Lanzhou University, Lanzhou, China; ^2^Department of Gynecology and Obstetrics, West China Second University Hospital, Sichuan University, Chengdu, China; ^3^State Key Laboratory of Biotherapy, West China Hospital, Sichuan University, and Collaborative Innovation Center of Biotherapy, Chengdu, China; ^4^The Department of General Surgery, The First Hospital of Lanzhou University, Lanzhou, China; ^5^Gansu Province Institute of Hepatopancreatobiliary Surgery, Lanzhou, China

**Keywords:** cinobufotalin, phosphoproteome, intrahepatic cholangiocarcinoma, DNA damage, apoptosis, ATM/CHK2/p53 signaling pathway

## Abstract

Intrahepatic cholangiocarcinoma (ICC) is a malignant tumor that originates from bile duct’s epithelial cells and is usually characterized by insidious symptoms and poor prognosis. Cinobufotalin (CB), an active ingredient obtained from the Traditional Chinese Medicine ChanSu, is purported to exhibit a wide range of antitumorigenic activities. However, the mechanism by which it achieves such pharmacological effects remains elusive. Here, we disclosed the mechanism of action by which CB inhibits ICC cells. Initial experiments revealed that the proliferation of RBE and HCCC-9810 cells was significantly inhibited by CB with IC50 values of 0.342 μM and 0.421 μM respectively. CB induced the expression of caspase-3 subsequently leading to the apoptosis of ICC cells. Phosphoproteomics revealed that the phosphorylation of many proteins associated with DNA damage response increased. Kinase-substrate enrichment analysis revealed that ATM was activated after CB treatment, while CDK1 was inactivated. Activated ATM increased p-CHK2-T68 and p-p53-S15, which promoted the expression of FAS, DR4 and DR5 and triggered cell apoptosis. In summary, this work reveals the role of CB in inducing DNA damage and cell apoptosis involved in the activation of the ATM/CHK2/p53 signaling pathway, and indicates that CB may serve as a chemotherapeutic drug candidate for ICC treatment.

## Introduction

Cholangiocarcinoma may be classified as intrahepatic, perihilar, and distal according to its anatomical location in relation to the biliary tree. Intrahepatic cholangiocarcinoma (ICC), located above the confluence of the left and right hepatic ducts, is a malignant tumor originating from biliary epithelial cells and is characterized by a high degree of malignancy, insidious symptoms, and poor prognosis (1). The incidence of ICC has increased rapidly over the past few decades (2–4), with the highest incidence in Thailand at approximately 0.096% in males (5). The pathogenic factors and pathogenesis of cholangiocarcinoma are still inconclusive. A recent meta-analysis has showed that bile duct cysts, gallstones, liver cirrhosis, and hepatitis B and C viruses are risk factors for intrahepatic and extrahepatic cholangiocarcinoma (6). At present, the main strategies for cholangiocarcinoma treatment are surgical resection and chemotherapy (7). However, current chemotherapy drugs are limited in efficacy and prone to resistance. Patients with locally advanced or metastatic cholangiocarcinoma receiving standard chemotherapy regimens (gemcitabine and cisplatin) have a median overall survival of less than one year (8). Emerging targeted drugs, such as FGFR inhibitors, IDH inhibitors and PD-1 monoclonal antibodies, have become promising (7, 9), but their clinical applications are limited due to the high prices and restricted applicable population. Therefore, it is still urgent to explore new, safe, universal and effective anti-cholangiocarcinoma drugs.

Traditional Chinese Medicine, included some chemotherapeutic compounds, has received increased attention. Cinobufotalin (CB), an active ingredient of the Traditional Chinese Medicine ChanSu, is a bufadienolide derived from toad venom (10). A two-phase solvent system, consisting of hexane/ethyl acetate/methanol/water at 4:6:2:4 V/V, 4:6:2.5:4 V/V and 4:6:3.2:4 V/V, was used to separation and purification of cinobufotalin from ChanSu, based on a high speed counter current chromatography (11). CB is known to have antitumorigenic activities and increase chemosensitivity (12–14). In addition, Cinobufotalin injection is widely used as an adjunctive anti-tumor therapy for lung cancer and hepatocellular carcinoma (15–18). However, the mechanism of action of CB remained elusive.

In this study, we found that CB inhibited ICC cell proliferation and promoted cell apoptosis. By using phosphoproteomics, we found significant changes in the proteome and phosphorylome associated with DNA damage and apoptosis in response to CB treatment. Further investigations demonstrated that CB induced ICC cell apoptosis by activating the ATM/CHK2/p53 signaling pathway and upregulating the expression of FAS, DR4 and DR5. Our results collectively indicate that CB may serve as a potential anti-cholangiocarcinoma drug.

## Methods

### Cell culture

The human ICC cell lines (RBE and HCCC-9810) were purchased from the National Biomedical Experimental Cell Resource Bank of China (Beijing, China). CB was purchased from Biopurify Phytochemicals Ltd (Chengdu, China). The ICC cells were grown in RPMI 1640 medium (#10270-106, Gibco) containing 10% (v/v) fetal bovine serum (#SFBE, NATOCOR), 100 U/mL penicillin and 100 μg/mL streptomycin (#15140-122, Gibco) and placed in an incubator with a constant temperature of 37°C and 5% CO_2_.

### Cell proliferation and colony formation assays

Cell proliferation ability was measured by the cell counting kit-8 (CCK-8). Cells were seeded in 96-well plates at a density of 3.5×10^3^ cells/well. After overnight incubation, the cells were treated with different concentrations of CB (0, 0.1, 0.25, 0.5, 1.0 and 2.0 μM) for 24 h, 48 h, and 72 h. Ten microliters of CCK-8 (Yeasen Biotechnology, Shanghai, China) was added, and the optical density (OD) value was detected at a wavelength of 450 nm after incubation at 37°C for 2 h. In the colony formation experiment, cells were seeded in 6-well plates for 2000 cells/well. Cells were treated with CB after sticking to the plates for 24 h, and the experiment was terminated after 2 weeks of culture. Clones were fixed with 4% paraformaldehyde for 30 minutes and stained with crystal violet for another 30 minutes.

### Apoptosis analysis by flow cytometry

Cells were treated with different concentrations of CB (0, 0.25 and 0.5 μM) for 48 h. According to the operating instructions, cells were harvested and washed twice with pre-cooled PBS, and stained with Annexin V-PE and 7-AAD solutions (Vazyme Biotech Co, Nanjing, China) at 25°C for 10 min in the dark. BD FACSCelestaTM multicolor flow cytometer (BD Bioscience, Franklin Lakes, NJ, USA) was used for detection. Annexin V-PE positive/7-AAD negative indicates early apoptotic cells, and Annexin V-PE positive/7-AAD positive means late apoptotic cells. All the above data were statistically analyzed in SPSS 20 (IBM, Chicago, IL, USA). Student’s *t*-test was used to compare two groups, and ANOVA was used for multiple groups. GraphPad Prism 8.0 (GraphPad, San Diego, CA, USA) was used for graphing.

### Protein extraction and TMT labeling

RBE cells treated with different concentrations (0, 0.25 μM) of CB for 48 h were harvested, washed twice with pre-cooled PBS, lysed in RIPA buffer (150 mM NaCl, 50 mM Tris (pH = 7.5), 1% NP-40 (v/v), 0.5% sodium deoxycholate (w/v)), 1×protease inhibitors, 1× phosphatase inhibitors), and sonicated with JY92-IIN sonicator (NingBoXinYi, Ningbo, China) for 3 min (parameters: on for 3 s, off for 10 s, power 30%). The protein extracts were centrifuged at 16000 g for 30 minutes. The protein concentration was determined with a BCA kit (Beyotime Biotech, Shanghai, China). One hundred micrograms of proteins from each sample were reduced with Tris (2-carboxyethyl) phosphine (TCEP) at a final concentration of 10 mM for 1 hour at 56°C, and alkylated with iodoacetamide at 20 mM in the dark. The proteins were then precipitated with methanol, chloroform and water (CH_3_OH : CHCl_3_:H_2_O = 4:1:3) and digested with trypsin (1:50, w/w, trypsin/protein) overnight at 37°C. The peptides of each sample were labeled with TMT (Thermo Fisher Scientific, Waltham, MA, USA) reagent according to the specification. After quenching with 5% hydroxylamine, the TMT-labeled peptides were mixed, and concentrated to dryness.

### Peptide fractionation

TMT-labeled peptides were desalted and then fractionated using reversed-phase high-performance liquid chromatography (RP-HPLC, SHIMADZU-LC-2030 Plus) under basic conditions. The mobile phase was composed of buffer A (ACN 98%, H2O 2%, pH = 10) and buffer B (90% ACN, H2O 10%, pH = 10). A standard 120 min LC gradient was used as follows: 0-2 min, 2% Buffer B; 2-60 min, 2-35% Buffer B; 60-95 min, 35-55% Buffer B; 95 -105 min, 55-90% Buffer B; 105~120 min, 90~2% Buffer B, and the flow rate was 1 ml/min. The mixture was separated into 120 fractions and combined into 20 parts. Then the samples were analyzed by mass spectrometry after desalting. For phosphorylomics, C18 solid-phase extraction cartridge (100 mg packing material) was used to divide the mixture into 18 fractions, which were finally combined into 6 parts and desalted.

### Enrichment of phosphopeptides

Twenty microliters of Fe-NTA agarose beads were used for the enrichment of phosphopeptides for each sample. The agarose beads were washed with 1 mL of washing buffer (80% ACN%, 0.1% TFA) 3 times and then transferred into six 1.5 mL EP tubes. Desalting peptides were resuspended in 300 µL washing buffer, and incubated with Fe-NTA agarose beads at room temperature for 45 minutes on a 3D shaker. Then the beads were washed with washing buffer 5 times and eluted with 150 µL elution buffer (50% ACN, 2.5% ammonia). The solution was neutralized with 8 µL 20%TFA, concentrated to dryness, and analyzed by mass spectrometry after desalting.

### Mass spectrometry analysis

Desalted peptides were resuspended in buffer A (2% ACN, 0.1% FA) and further analyzed by EASY-nanoLC 1000 coupled to a high-resolution mass spectrometer (Q Exactive Plus, Thermo Fisher Scientific). The samples were loaded onto a 75 μm (inner diameter) × 2 cm (length) trap column and a 75 μm (inner diameter) × 15 cm (length) analytical column, which were packed with C18 particles (DIKMA) in-house. Data-dependent acquisition (DDA) was performed in positive ion mode, and samples were analyzed with a 105 min or 65 min gradient from 12 or 13 to 100% buffer B (80% ACN, 0.1% FA) for proteomics and phosphoproteomics, respectively, at a flow rate of 330 nL/min. The full-scan MS spectrum ranged from 350 to 1600 m/z with a resolution of 70,000 at m/z = 200. The automatic gain control (AGC) value was set to 3e^6^, and the maximum injection time was 20 ms. In the MS/MS scan, the top 15 most abundant precursor ions were selected with an isolation window of 0.6 m/z. For the proteome, the collision energy (NCE) was set to 30%, but the phosphopeptides were fragmented with gradient energies of 25% and 31% NCE. The AGC value for MS/MS was set to 1e^5^, the maximum injection time was 100 ms, and the resolution was 35,000. Precursor ions with charge state z = 1, 7-8 or unassigned were excluded.

### Database search

The raw mass spectrometry files of the 20 fractions of the proteome and the 6 fractions of the phosphorylome were searched using Maxquant (version 1.6.2.3), referring to the Swiss-Prot human protein sequence database (20413 sequence, 2019/02/13 update), separately. The mass deviation of precursor ions was set to within 10 ppm, and the mass deviation of fragment ions was 0.02 Da. The maximum number of missing cleavage sites was 2. Cysteine carbamidomethylation and methionine oxidation were fixed modifications. For the phosphorylome, phosphorylation (S/T/Y) was set as a dynamic modification. The false discovery rate of peptides and proteins was less than 1%. The minimum number of amino acids in a peptide is 6, and the maximum molecular weight of a peptide is 12,000 Da.

### Proteome data processing

After removing reverse and contaminant proteins, the total protein intensities of all samples were normalized, and proteins with unique peptides >= 2 were used for subsequent data analysis. The phosphosites with localization probabilities greater than 0.75 were retained. Proteins and phosphosites with *p* < 0.05 (Student’s *t*-test) and ratio (CB/Ctr) > 1.25 or < 0.8 were defined as significantly changed after CB treatment. The algorithm for summarizing the change in total phosphorylation (ΔP) at different sites on the same protein is as follows: the ΔPs value of each phosphoprotein is equal to the “sum of log2(fold change)” of all significantly changed phosphopeptides of protein isoforms encoded by the same gene (19).

### Bioinformatics

Kyoto Encyclopedia of Genes and Genomes (KEGG) enrichment analysis of significantly differentially expressed proteins was performed by DAVID 6.8 (https://david.ncifcrf.gov/home.jsp) (20, 21). Gene function enrichment and molecular network interaction analysis of differentially phosphorylated proteins were carried out by Metascape (https://metascape.org) (22). Combined analysis of quantified proteomic and phosphoproteomic data with literature-based proteome-wide signaling networks was based on the SIGnaling Network Open Resource 2.0 Database (SIGNOR 2.0) (https://signor.uniroma2.it) (23). Data processing and visualization were performed using the following R packages: ComplexHeatmap, ggplot2, ggpubr, ggthemes, dplyr, ggExtra, and readxl.

### Immunoblotting

Cells were treated with CB (0, 0.25, 0.5 μM) for 48 h and then the cell lysates were analyzed by immunoblotting. The primary antibodies used in this study were as follows: ATM antibody (ET1606-20, Huabio), phospho-ATM-S1981 antibody (ET1705-50, Huabio), phospho-AKT-S473 antibody (4060S, Cell Signaling Technology), AKT antibody (4685S, Cell Signaling Technology), phospho-mTOR-S2448 antibody (5536T, Cell Signaling Technology), mTOR antibody (2983T, Cell Signaling Technology), phospho-p70S6K-T389 antibody (9234S, Cell Signaling Technology), p70S6K antibody (9202S, Cell Signaling Technology), Bcl-2 antibody (ET1702-53, Huabio), active+pro Caspase-3 antibody (ET1608-64, Huabio), active Caspase-3 antibody (ET1602-47, Huabio), p53 antibody (ab26, Abcam), γH2AX antibody (201082-2A9, ZEN-BIOSCIENCE), phospho-p53-S15 antibody (YP0205, ImmunoWay), CHK2 antibody (ET1610-52, Huabio), phospho-CHK2-T68 antibody (29012-1-AP, Proteintech) and GAPDH antibody (60004-1-lg, Proteintech). The relative protein intensity was calculated by ImageJ software.

## Results

### CB inhibits ICC cell proliferation and promotes apoptosis

**Figure 1:**
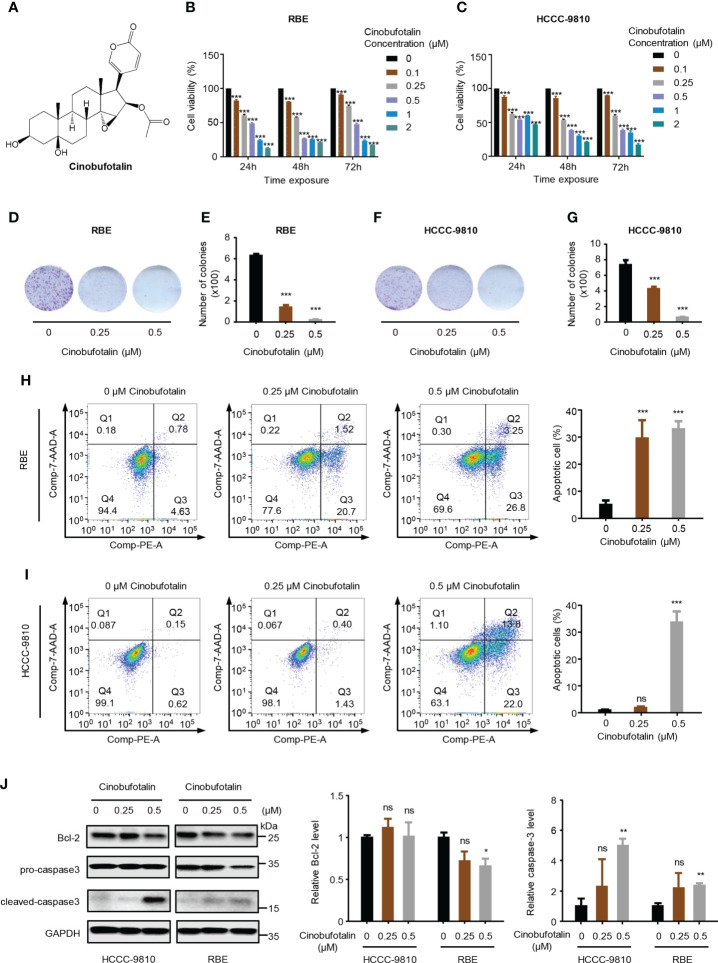
Cinobufotalin inhibits ICC cell proliferation and promotes cell apoptosis. **(A)** Chemical structure of cinobufotalin (CB). **(B, C)** RBE and HCCC-9810 cells were treated with 0, 0.1, 0.25, 0.5, 1.0 and 2.0 μM of CB, respectively, for 24 h, 48h and 72h, and cell viability was measured by Cell counting kit-8 (CCK-8) assay. **(D-G)** Colony formation **(D, F)** and the statistics of colony counts **(E, G)** of REB and HCCC-9810 cells. **(H-I)** Flow cytometry analysis of cell apoptosis by Annexin V-PE/7-AAD staining. **(J)** The expression of apoptosis-related proteins in RBE and HCCC-9810 cells measured by immunoblotting and calculated by ImageJ software. *p < 0.05, **p < 0.01, ***p < 0.001, *ns* denotes statistically insignificant difference, Student’s *t*-test.

The chemical structure of CB is shown in **Figure 1A**. CB significantly inhibited the proliferation of ICC cells, including RBE and HCCC-9810 cells. The IC50 values of CB in RBE and HCCC-9810 cells were 0.342 μM and 0.421 μM, respectively (**Figures 1B, C**). Meanwhile, we found that application of 0.5 μM cinobufotalin in RBE cells shows higher cell viability at 72 h than 48 h post-treatment (**Figure 1B**), which may be related to the cell contact inhibition over longer periods of treatment in the untreated group for the too high cell density. Therefore, the calculated relative cell viability may be slightly higher at the timepoint of 72 h than at 48 h. In addition, CB also significantly inhibited the colony formation of ICC cells (**Figures 1D–G**). Flow cytometry analysis revealed dramatically increased numbers of early and late apoptotic RBE and HCCC-9810 cells as the concentrations of CB increased (**Figures 1H, I**). Consistently, immunoblotting showed that the cleaved caspase-3, an apoptosis-associated protein marker, was up-regulated in both ICC cell lines, and the oncoprotein Bcl-2 was down-regulated in RBE cells after CB treatment (24–26) (**Figure 1J**). Collectively, the above results indicate that CB can inhibit ICC cell proliferation and induce cell apoptosis.

### Phosphoproteomics reveals CB-regulated signaling pathway

**Figure 2:**
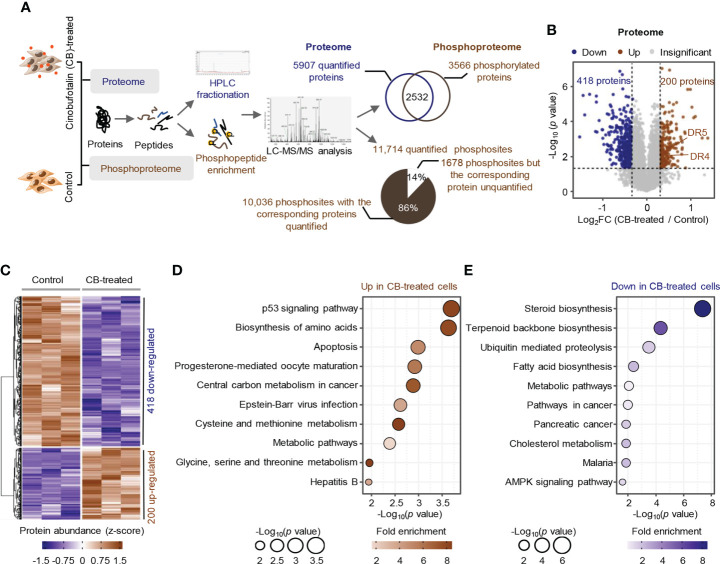
Proteome profile of CB-treated RBE cells. **(A)** The workflow for the identification and quantification of proteins and phosphosites. **(B, C)** Volcano plot **(B)** and heatmap **(C)** of differentially expressed proteins (*p* < 0.05 (Student’s *t*-test) and ratio (CB/Ctr) > 1.25 or < 0.8). Brown represents upregulated, blue means downregulated. **(D, E)** KEGG pathway analysis of significantly upregulated proteins **(D)** and downregulated proteins **(E)**. Circle size indicates the *p* value of enrichment, and the color shows the fold enrichment.

To profile the CB-induced molecular changes in ICC cells, we performed comparative proteomic and phosphoproteomic analyses between CB-treated and untreated RBE cells with 3 biological replicates (**Figure 2A**). A total of 6740 proteins were identified by proteomics, and 5907 were quantified with unique peptides >= 2 (**Supplemental Table S1**). Among them, 200 proteins were significantly up-regulated after CB treatment (ratio (CB/Ctr) > 1.25, *p* < 0.05), and 418 proteins were significantly down-regulated (ratio (CB/Ctr) < 0.8, *p* < 0.05) (**Figure 2B** and **Supplemental Table S2**). Among the up-regulated proteins, DR4 (also known as TNFRSF10A and TRAIL-R1) and DR5 (also known as TNFRSF10B and TRAIL-R2) are the two main types of tumor necrosis factor-related apoptosis-inducing ligand (TRAIL) receptors in humans and can induce cell apoptosis (27, 28) (**Figures 2B, C**). For example, in Hep3B hepatoma cells, morin up-regulates DR4 and DR5, and activates caspase-3, -8, and -9 to promote tumor cell apoptosis (29). In addition, DNA damage can induce the expression of DR5 through a p53-dependent pathway, which further promotes tumor cell apoptosis (30). KEGG enrichment analysis showed that the up-regulated proteins were mainly associated with p53, biosynthesis of amino acids and apoptosis signaling pathways and amino acid metabolism, such as cysteine and methionine metabolism and glycine, serine and threonine metabolism (**Figure 2D**). The down-regulated proteins were involved in steroid biosynthesis, terpenoid biosynthesis, ubiquitin mediated proteolysis, fatty acid biosynthesis and cholesterol metabolism, as well as the AMPK signaling pathway and tumor-related signaling pathway (**Figure 2E**).

**Figure 3:**
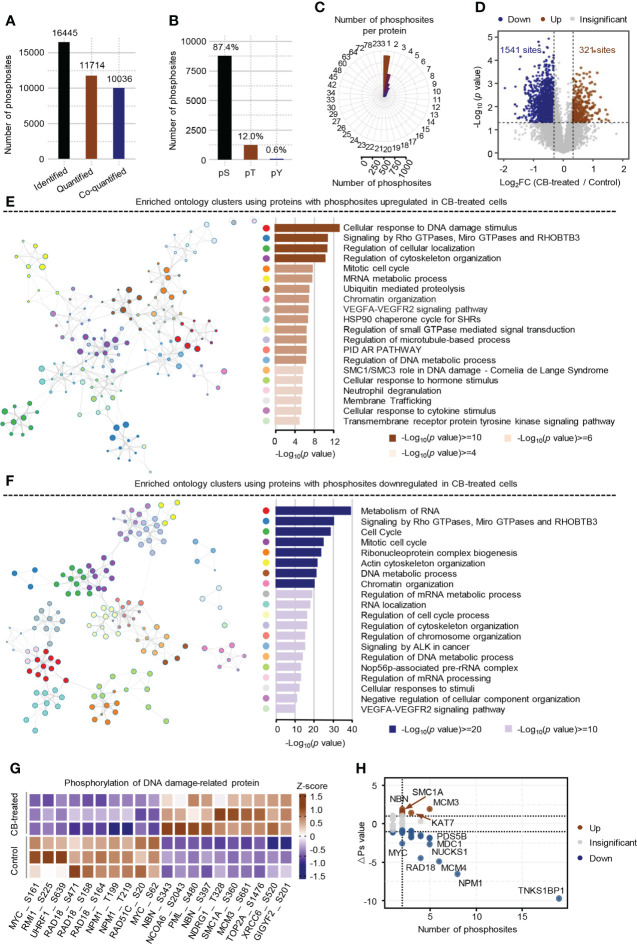
Phosphoproteome profile of CB-treated RBE cells. **(A)** The statistics of identified and quantified phosphosites. Co-quantified means both phosphosites and the corresponding proteins were quantified. **(B)** The percentages of phosphosites on serine, threonine and tyrosine residues. **(C)** The statistics of the number of phosphosites on each protein. **(D)** Volcano Plot of significantly altered phosphosites (*p* < 0.05 (Student’s *t*-test) and ratio (CB/Ctr) > 1.25 or < 0.8). Brown represents up-regulated, blue means down-regulated. **(E, F)** Pathway enrichment analysis of proteins with up-regulated **(E)** and down-regulated **(F)** phosphosites using Metascape. The colors of the dots in the network represents different biological pathways. **(G)** Heatmap of the top 10 DNA damage-related phosphosites that were significantly changed in CB-treated versus untreated cells. **(H)** Global ΔPs analysis of phosphoproteins involved in DNA damage. Brown represents up-regulated, blue means down-regulated.

Phosphorylomics identified a total of 16,445 phosphosites, of which 11,714 were quantified with phosphorylation localization probability greater than 0.75. Of the 11,714 quantified phosphosites, the corresponding proteins of 10036 phosphosites were quantified as well and were used for the subsequent analysis (**Figure 3A**). Consistent with previous mass spectrometry-based analyses (31, 32), the identified phosphorylation events in ICC cells mainly occurred on serine residues (87.4%) (**Figure 3B**) and most proteins had fewer than 3 identified phosphosites (**Figure 3C**). The changes of the 10036 phosphosites between CB-treated and untreated RBE cells were calculated and normalized to protein intensities (**Supplement Table S3**). As a result, 321 up-regulated phosphosites (ratio (CB/Ctr) > 1.25, *p* < 0.05) and 1541 down-regulated phosphosites (ratio (CB/Ctr) < 0.8, *p* < 0.05) were obtained (**Figure 3D** and **Supplement Table S4**). Although the signaling pathways including signaling by Rho GTPases, Miro GTPases and RHOBTB3 were enriched using proteins with up- or down-regulated phosphosites (**Figures 3E, F**), we still observed many differences between them. For example, pathways such as cellular response to DNA damage stimulus and regulation of cellular localization were enriched using proteins with up-regulated phosphosites (**Figure 3E**), while metabolism of RNA and cell cycle were enriched using proteins with down-regulated phosphosites (**Figure 3F**).

Further analysis showed that 176 significantly differential phosphosites on 84 proteins were associated with DNA damage. Some well-known DNA damage-related phosphosites, such as NBN-S343/397, MCM3-S681 and MYC-S62 were significantly changed after CB treatment (**Figure 3G**) (33–36). To obtain the DNA damage associated proteins with the greatest changes in total phosphorylation, we summed the phosphorylation alteration for each protein based on a known algorithm (**Supplement Table S5**) (19). The results showed that the total phosphorylation levels of NBN, MCM3, SMC1A and KAT7 were significantly increased, while those of TNKS1BP1, NPM1, MCM4, RAD18, NUCKS1, MYC and many other proteins were significantly decreased (**Figure 3H**). Collectively, CB may inhibit cell proliferation by inducing DNA damage and apoptosis.

### Kinase-substrate enrichment analysis reveals the activation of ATM by CB

**Figure 4:**
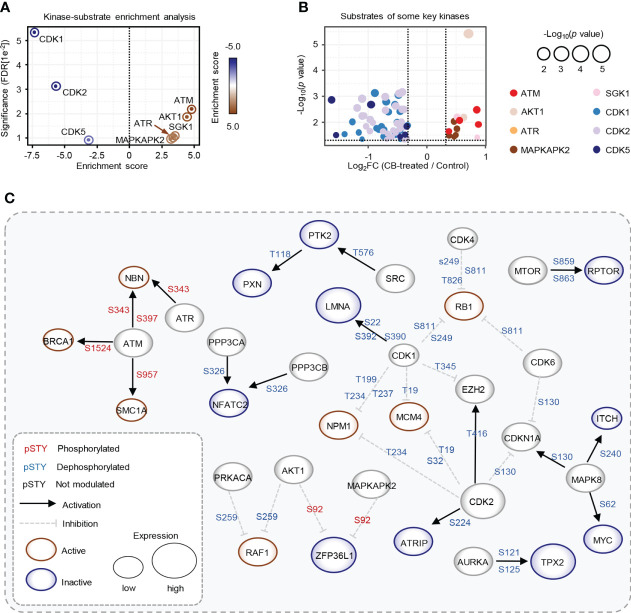
Key kinases involved in CB treatment of RBE cells. **(A)** Evaluation of kinase activities by KSEA in CB-treated RBE cells. **(B)** Scatter plot of the significantly changed substrates of key kinases. The color represents different substrates of kinases that showed in Figure 4A. **(C)** The phosphoproteome alterations in response to CB treatment were mapped to a literature derived signal network using the SIGNOR 2.0 database. The node size is proportional to the level of protein expression. Blue and red nodes mean inactivated and activated, respectively. Phosphorylation and dephosphorylation reactions on specific residues are represented by edges between nodes.

We further used the PhosphoSitePlus database in the KSEA APP website to perform a kinase-substrate enrichment analysis based on the 10,036 phosphosites (37, 38), with an FDR cutoff of 0.05 and a substrate count cutoff of 5, and found that ATM kinase substrate motifs, as well as the substrate motifs of other kinases, such as AKT1, ATR, SGK1 and MAPKAPK2, were significantly enriched (**Figure 4A**), with the phosphorylation levels of ATM, AKT1, ATR, SGK1 and MAPKAPK2 substrates increased after CB treatment (**Figure 4B**). In addition, we found that the substrate motifs of cell cycle-related kinases such as CDK1/2/5 were also enriched (**Figure 4A**), and the phosphorylation levels of CDK1/2/5 substrates decreased after CB treatment (**Figure 4B**).

To further explore intracellular changes in signaling networks after CB treatment, we performed integrative analysis of the proteome and phosphoproteome data using the SIGNOR 2.0 database (23, 32, 39). The results showed that the downstream substrates of ATM were hyperphosphorylated in RBE cells after CB treatment, while CDK1 and CDK2 substrates were significantly dephosphorylated (**Figure 4C**). ATM is activated with increased phosphorylation at S1981 and acetylation at K3106 and then initiates the signaling cascade in response to DNA damage (40, 41). Although phosphorylation of ATM at S1981 was not detected in our phosphoproteomic data, we speculated that CB might activate ATM based on the activation of its numerous downstream substrates.

### CB promotes apoptosis by activating the ATM/CHK2/p53 signaling pathway

**Figure 5:**
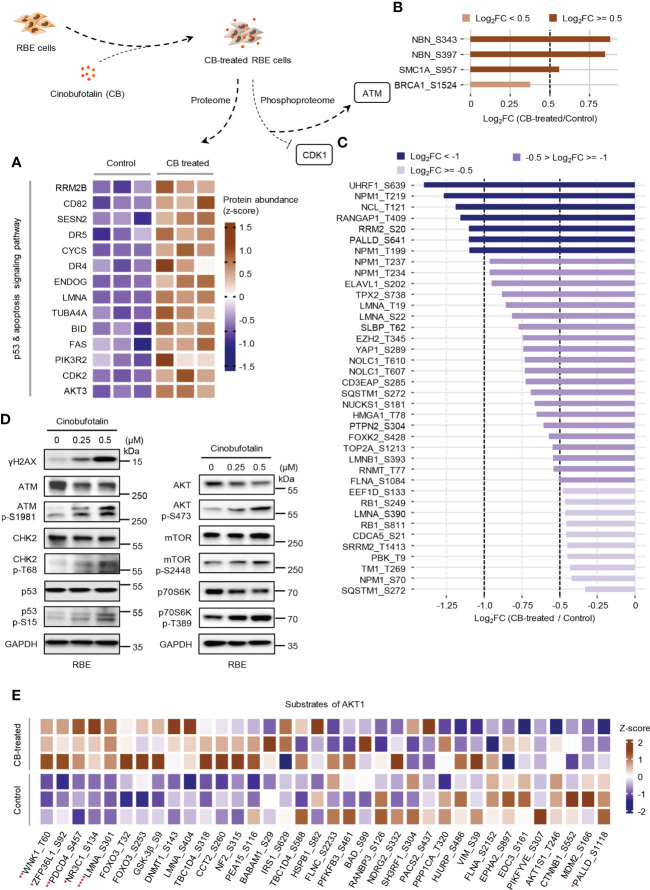
CB treatment remodels proteome and phosphoproteome of RBE cells. **(A)** Heatmap of significantly changed proteins of the p53 and apoptosis signaling pathway in CB-treated RBE cells. Brown represents up-regulated, blue means down-regulated. **(B, C)** The statistics of the phosphosites regulated by ATM **(B)** and CDK1 **(C)**. **(D)** Verification of the expression of DNA damage, p53 signaling and AKT signaling pathway-related proteins and phosphosites by immunoblotting. **(E)** The heatmap shows the MS detected phosphosites regulated by AKT1. *p < 0.05, **p < 0.01, ****p < 0.0001, Student’s *t*-test.

**Figure 6:**
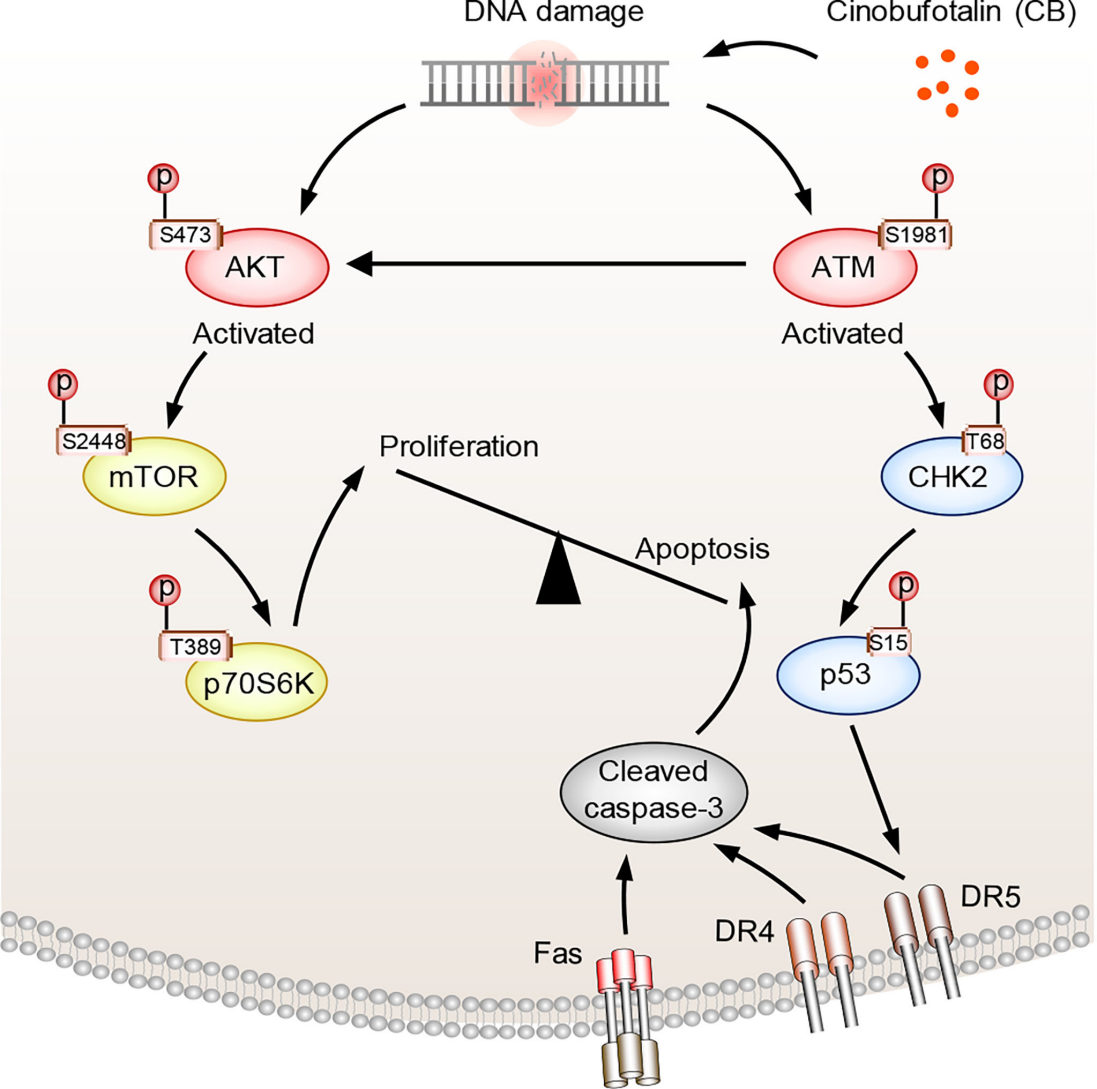
The proposed mechanism of action of CB in ICC cells. CB treatment induces DNA damage and activates ATM and AKT. Activated ATM increases p-CHK2-T68 and p-p53-S15, which promotes the expression of FAS, DR4 and DR5 and triggers cell apoptosis. Activated AKT, meanwhile, promotes cell survival by activating mTOR and its downstream signaling pathway. However, the tumor cells underwent apoptosis after CB treatment due to the predominant activation of the ATM/CHK2/p53 apoptotic pathway.

In the above studies, we found that CB induced significant up-regulation of key proteins involved in the p53 and apoptosis signaling pathways in RBE cells (**Figure 5A**). The phosphorylation levels of ATM substrates, including NBN-S343/397, SMC1A-S957 and BRCA1-S1524, were up-regulated (**Figure 5B**), while the phosphorylation levels of CDK1 substrates, such as UHRF1-S639, NPM1-T219 and NCL-T121, were reduced (**Figure 5C**). Activated ATM can stimulate CHK2 by promoting phosphorylation at CHK2-T68, which stabilizes and activates p53 by promoting phosphorylation at p53-S15/S20 (42–44). When p53 is activated, cell apoptosis can be induced by up-regulating the FAS, DR4 and DR5 signaling pathways (45–49). Notably, our proteomic data showed that FAS, DR4 and DR5 were up-regulated after CB treatment (**Figure 5A**). Moreover, the levels of γH2AX, p-ATM-S1981, p-CHK2-T68, and p-p53-S15 were also significantly increased in CB-treated cells (**Figure 5D**). Previous studies have demonstrated that the activation of the ATM/CHK2/p53 signaling pathway can inhibit tumor cell growth by inducing apoptosis and cell cycle arrest (42, 50–54). In addition, we found that AKT1 was also activated after CB treatment. In order to further show the changes of AKT1-associated signaling pathways, we summarized AKT1 substrates (**Figure 5E**). Although some downstream substrates of AKT1 such as FOXO3 and GSK3β showed increased phosphorylation levels, but the changes were not significant. To further demonstrate our results, we performed Western blotting analysis of AKT1 phosphorylation and its downstream mTOR signaling (AKT Ser473, mTOR S2448 and p70S6K T389), and found that the phosphorylation level of both sites increased, indicating the activation of AKT/mTOR/p70S6K signaling pathway, which might activate pro-survival AKT signaling after DNA damage (**Figure 5D**) (55). However, the tumor cells underwent apoptosis after CB treatment due to the predominant activation of the ATM/CHK2/p53 apoptotic pathway. Therefore, this work confirms that CB induces DNA damage, activates the ATM/CHK2/p53 signaling pathway and promotes the expression of FAS, DR4 and DR5, thereby promoting apoptosis in ICC cells and ultimately inhibiting cell proliferation (**Figure 6**).

## Discussion

As a potential anti-cancer drug, some mechanisms of action of CB have been proposed by previous studies. For example, CB inhibits cancer cell stemness and EMT signaling by regulating the PI3K/AKT/MYC/p53/miR-133a-3p signaling axis (56). It can induce reactive oxygen species (ROS) production and FAS up-regulation, which in turn leads to DNA damage and caspase-dependent apoptosis (12, 13). However, a comprehensive understanding of the mechanisms by which CB induces DNA damage, apoptosis and cell cycle arrest is still lacking. This study revealed that CB inhibits ICC cell proliferation through the ATM/CHK2/p53 signaling pathway, providing a new perspective for understanding the anti-tumor mechanism of CB.

The MRN complex (MRE11/RAD50/NBN) is a DNA double-strand break sensor in eukaryotes involved in the early steps of DNA double-strand break sensing and repair, DNA excision, activation of DNA damage checkpoints, activation of cell cycle checkpoints, chromatin remodeling, and recruitment of DNA repair factors (57–59). NBN (NBS1) is a component of the MRN complex and has many serine residues that can be phosphorylated. Among them, S343, S397 and S615 can be phosphorylated by ATM, and ATM-mediated phosphorylation of NBN is essential for the DNA damage response (34). In addition, radiation-induced DNA damage can lead to increased phosphorylation levels of MCM3 at S681/S728/S734 (35). Consistently, our results showed that CB treatment of RBE cells leads to DNA damage and up-regulation of phosphorylation at both NBN-S343/397 and MCM3-S681, confirming the role of CB in inducing DNA damage.

We also observed significant dephosphorylation events at S62 and S161 of MYC in our phosphoproteomics analysis after CB treatment. As a classic oncogenic factor, MYC, a downstream regulator of the PI3K/AKT signaling pathway, can negatively regulate p53 (56, 60). Dephosphorylation of MYC at S62 can lead to decreased MYC activity and protein stability, thereby inhibiting cell proliferation (36, 61). Therefore, we assume that MYC may serve as an effector molecule to promote the function of CB in inhibiting ICC cell proliferation.

TNKS1BP1 is a member of the poly ADP ribose polymerase (PARP) superfamily protein associated with DNA damage repair. Overexpression of TNKS1BP1 induces autophosphorylation of DNA-PKcs at S2056, thereby increasing DNA damage repair ability (62). In this study, we found that the phosphorylation level of TNKS1BP1 was significantly decreased after CB treatment, which may be associated with CB induced DNA damage. Moreover, ubiquitination is an important post-translational modification of proteins, which plays a crucial role in maintaining cell function (63). We also noticed that dephosphorylation events occurred on some serine residues of E3 ligases, such as RAD18 at S158/S164/S471 and RAD51C at S20 after CB treatment. RAD18 and RAD51 are DNA damage repair factors and participate in DNA damage repair (64–66). The roles of phosphorylation of RAD18 and RAD51 in DNA damage repair are less understood and worthy of further exploration.

Although the mechanism of CB has been disclosed, this study also had some limitations. First, the function of CB in inhibiting cancer cell proliferation needs to be verified in animal models, and the effects of CB on normal organs require a comprehensive evaluation. Second, the direct cellular targets of CB are still unknown. CB has an epoxy group, which may react with some nucleophilic groups in proteins and other biological macromolecules and initiate the DNA damage process. In addition, CB is a cardiotonic steroids or bufadienolides. The structure of CB is very close to that of cholesterol and other steroid derivatives. According to the pathway enrichment result, we assume that cinobufotalin may competitively bind to the receptor of these steroids and affect the steroid biosynthesis. However, this assumption needs to be verified with more experiments.

## Conclusion

Collectively, in this study, we investigated the function of CB in ICC cells by performing phosphoproteomics. Several findings were made. First, proteomic analysis showed that the p53 and apoptosis signaling pathways were activated after CB treatment. The proteins in these signaling pathways, such as DR4, DR5, FAS, CYCS, ENDOG, RRM2B, TUBA4A and SESN2, were significantly up-regulated, and the changes in some classical markers among them were further verified. Second, phosphoproteomics revealed the activation of some DNA damage-associated kinases after CB treatment, such as ATM, AKT1 and ATR, while the cell cycle-related kinases CDK1/2/5 were inactivated. Finally, we demonstrated that CB promoted apoptosis in ICC cells and inhibited cell proliferation by activating the DNA damage associated ATM/CHK2/p53 signaling pathway and promoting the expression of FAS, DR4 and DR5. In summary, this work preliminarily reveals the mechanism of action of CB and provides a potential chemotherapeutic drug candidate for cholangiocarcinoma treatment.

## Data availability statement

The datasets presented in this study can be found in online repositories. The names of the repository/repositories and accession number(s) can be found in the article/**Supplementary Material**.

## Author contributions

WM and YL designed the experiment; ZX, ML, and MH handled the sample preparation and data analysis; ZX drafted and revised the manuscript; the remaining authors provided technical support and suggestions. All authors have approved the final version of the manuscript.

## Funding

This work was supported by Grant(s) from the National Natural Science Foundation of China (82060551 to WM), the Health Industry Scientific Research Program of Gansu Province (GSWSKY2020-11 to YYL), the Lanzhou Cheng guan District Science and Technology Plan Project (2019JSCX0092 to YYL) and the Key Talent Program of Gansu Province (2019RCXM077 to WM).

## Conflict of interest

The authors declare that the research was conducted in the absence of any commercial or financial relationships that could be construed as a potential conflict of interest.

## Publisher’s note

All claims expressed in this article are solely those of the authors and do not necessarily represent those of their affiliated organizations, or those of the publisher, the editors and the reviewers. Any product that may be evaluated in this article, or claim that may be made by its manufacturer, is not guaranteed or endorsed by the publisher.
